# *LAMB2* p.E991K Mutation-Mediated Atherosclerosis in Rabbit

**DOI:** 10.3390/genes17070820

**Published:** 2026-07-18

**Authors:** Ronghan Zhang, Chao Mu, Shujun Yang, Liqiang Jiang

**Affiliations:** Key Lab for Zoonosis Research, College of Veterinary Medicine, Jilin University, Changchun 130062, China; zhangrh9922@mails.jlu.edu.cn (R.Z.); muchao24@mails.jlu.edu.cn (C.M.);

**Keywords:** atherosclerosis, LAMB2, genetic susceptibility, CRISPR/Cas9-SpG, NLRP3 inflammasome, rabbit

## Abstract

**Background**: Atherosclerosis is a major pathological basis of cardiovascular disease and is influenced by both genetic and environmental factors. Although genome-wide association studies have identified the *LAMB2* p.E987K variant as a susceptibility locus for atherosclerosis, its functional role remains unclear. **Methods**: In this study, a rabbit model carrying the homologous *LAMB2* p.E991K mutation was generated using the CRISPR/Cas9-SpG system and subjected to a high-fat diet to induce atherosclerosis. **Results**: *LAMB2* mutant rabbits exhibited increased body weight and significant lipid metabolic abnormalities. Oil Red O staining demonstrated enhanced lipid accumulation and larger atherosclerotic plaques in the aorta. In addition, α-SMA expression was reduced, whereas CD4 and MCP-1 expression was elevated, suggesting vascular smooth muscle cell loss and altered immune responses. Laminin β2 (LAMB2) expression was markedly decreased and accompanied by basement membrane disruption. Furthermore, activation of the NLRP3 inflammasome was observed in mutant rabbits. **Conclusions**: These findings demonstrate that the *LAMB2* p.E991K mutation promotes HFD-induced atherosclerosis by impairing basement membrane integrity, enhancing inflammation, and disrupting lipid metabolism, highlighting *LAMB2* as a genetic modifier of atherosclerosis susceptibility.

## 1. Introduction

Atherosclerosis is a complex chronic disease of the arterial wall involving dysregulated lipid metabolism, persistent inflammatory activation, and progressive vascular remodeling. The condition underlies the majority of cardiovascular events and represents the principal pathological basis of coronary artery disease, ischemic stroke, and peripheral arterial disease [1,2,3,4,5]. Although established risk factors such as dyslipidemia, hypertension, diabetes mellitus, and cigarette smoking play roles in the development and progression of atherosclerosis, they do not fully account for the interindividual variability in disease susceptibility, mounting evidence indicates that genetic factors are also important determinants of individual susceptibility to the disease [6,7,8,9,10]. Therefore, identifying novel susceptibility genes and elucidating their biological functions are essential for a comprehensive understanding of the molecular mechanisms underlying atherosclerosis.

Laminin β2 (LAMB2) is an essential constituent of the basement membrane, belonging to the laminin family of extracellular matrix proteins that are assembled from α, β, and γ chains through disulfide-bond interactions. It plays indispensable roles in maintaining tissue architecture, mediating cell adhesion, and regulating intracellular signaling pathways [11]. Recent genetic evidence from a GWAS of more than one million individuals demonstrated that the *LAMB2* variant rs34759087 (p.E987K) is significantly associated with atherosclerotic cardiovascular disease susceptibility [12]. Previous studies have demonstrated that mutations in *LAMB2* impair the synthesis and secretion of LAMB2, leading to defects in endothelial cell function and basement membrane assembly [13]. Furthermore, Wagner et al. reported that LAMB2 is highly expressed in vascular endothelial cells of young hearts but is markedly reduced during aging, thereby influencing endothelial cell phenotype and function [14]. In addition, pathogenic variants in *LAMB2* are recognized as the major genetic determinant of Pierson syndrome, a disorder characterized by basement membrane abnormalities and dyslipidemia, suggesting a potential role for LAMB2 in vascular homeostasis and lipid metabolism [15].

Despite these observations, the biological function of LAMB2 in atherosclerosis remains largely unexplored, and whether it directly contributes to disease development and progression has yet to be determined. Therefore, in the present study, we generated a homologous *LAMB2* p.E991K gene-edited rabbit model using the CRISPR/Cas9-SpG genome-editing system. Combined with high-fat diet induction, we systematically evaluated the effects of the *LAMB2* mutation on atherosclerotic phenotypes and investigated its potential molecular mechanisms. Our findings provide experimental evidence supporting the role of *LAMB2* as a genetic susceptibility factor in atherosclerosis and establish a large-animal model for investigating gene–environment interactions in vascular disease.

## 2. Materials and Methods

### 2.1. Rabbit and Ethical Statement

This study utilized New Zealand White rabbits (6–8 months, 3–5 kg) housed under standard controlled conditions at Jilin University’s Experimental Animal Center. The Animal Welfare and Ethics Committee of Jilin University approved all experimental protocols, which strictly adhered to established guidelines for the care and ethical use of laboratory animals (SY202309032).

### 2.2. Vector Construction and Transcription

For the construction of the *LAMB2* p.E991K rabbit model, two specific sgRNAs were designed and inserted into the BbsI site of a pUC57-T7-sgRNA backbone. T7-driven in vitro transcription was performed to produce the sgRNAs, which were subsequently purified using miRNeasy Mini kits (Qiagen, Hilden, Germany). For base editing, NotI-linearized SpG-BE4max plasmid served as the template for capped mRNA synthesis using the HiScribe T7 ARCA kit (NEB, MA, USA). The transcribed mRNA was purified prior to embryo microinjection.

### 2.3. Embryo Transfer

Following established protocols for pronuclear-stage embryo microinjection, a mixture of SpG-BE4max mRNA (120 ng/μL) and sgRNA (50 ng/μL) was co-injected into the cytoplasm of fertilized rabbit zygotes. These microinjected embryos were subsequently transferred into the oviducts of synchronized pseudopregnant recipients to develop to term.

### 2.4. Genotyping and Sanger Sequencing

Ear tissue samples collected from newborn rabbits were used for genomic DNA extraction. Genotypes were determined through PCR amplification of the *LAMB2* p.E991K target region followed by direct Sanger sequencing. The primer pairs employed for amplification were as follows:

F: 5′-AGCGGCACTTTGCTACTTCT-3′

R: 5′-CACCAGTCAAGAGGAGCCTG-3′

### 2.5. High-Fat Diet Induction

A rabbit diet supplemented with 2% cholesterol was purchased from Shuyu Biotechnology Co., Ltd (Shanghai, China). To induce atherosclerosis, rabbits were fed the high-cholesterol diet for four consecutive weeks. Animals were divided into four groups: the wild-type normal diet (WT-ND) group, the *LAMB2* normal diet (*LAMB2*^E991K^-ND) group, the wild-type high-fat diet (WT-HFD) group, and the *LAMB2* high-fat diet (*LAMB2*^E991K^-HFD) group. The sex of rabbits was not used as a selection criterion and was randomly determined based on availability. Blood samples were collected every two weeks throughout the induction period for dynamic monitoring of serum lipid profiles.

### 2.6. Measurement of Serum TC, TG, LDL-C, and HDL-C Levels

Centrifugation of whole blood collected from the rabbits was performed to obtain serum samples. Standard lipid profile parameters—namely total cholesterol (TC), triglycerides (TG), and high- and low-density lipoprotein cholesterol (HDL-C and LDL-C)—were subsequently quantified using an automated biochemical analyzer (Changchun, China) following the manufacturer’s instructions.

### 2.7. Histological and Immunohistochemical Analyses

The aortic arches of *LAMB2* mutant and WT rabbits were excised for histopathological analysis [16]. Tissue samples were fixed in 4% paraformaldehyde, embedded in paraffin wax, and cut into serial sections. PAS staining was then carried out to examine structural alterations of the vascular basement membrane [17]. Oil Red O staining was conducted on frozen sections according to a standard protocol to evaluate lipid deposition within the vascular wall [18].

Immunohistochemical (IHC) staining was conducted following standard procedures [19]. For IHC staining, we followed an established procedure that included tissue slide pretreatment (peroxidase quenching, thermal antigen retrieval, and serum blocking) prior to antibody incubation. Target proteins were probed with primary antibodies overnight, followed by a 1h incubation with Bio-Sheep secondary antibodies (1:1000). Finally, we utilized DAB and hematoxylin for signal visualization and nuclear counterstaining, respectively.

### 2.8. Western Blot Analysis

Total protein from aortic tissues was isolated using RIPA buffer containing 0.01% PMSF, separated via 12% SDS-PAGE, and transferred onto PVDF membranes. Following a blocking step with 5% non-fat milk, membranes underwent overnight primary antibody incubation at 4 °C. Next, HRP-conjugated secondary antibodies (1:1000, Beyotime, Shanghai, China) were applied. Finally, bands were detected using the ECL Plus system and their densities were analyzed with ImageJ (NIH, version 1.50i).

### 2.9. Statistical Analysis

All data (mean ± SD) were processed via GraphPad Prism 8.0.0, utilizing Student’s *t*-test or one-way ANOVA where appropriate. A *p*-value threshold of <0.05 was set for significance. Different levels of statistical significance are designated as follows: * *p* < 0.05, ** *p* < 0.01, *** *p* < 0.001, and **** *p* < 0.0001.

## 3. Results

### 3.1. Successful Generation of the LAMB2 p.E991K Gene-Edited Rabbit Model

**Figure 1:**
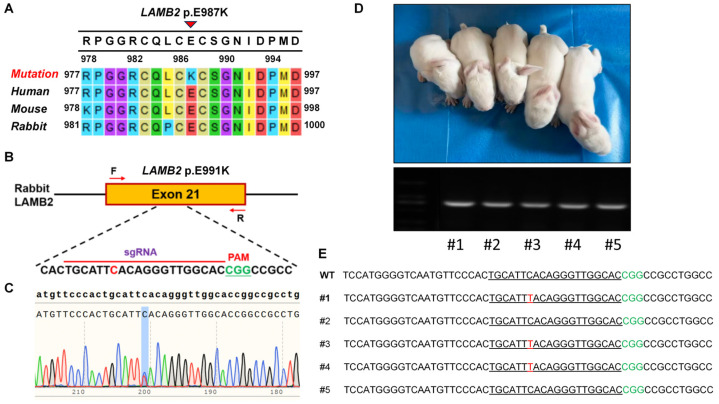
Generation of the *LAMB2* p.E991K mutant rabbit model. (**A**) Conservation analysis of the human mutation site across different species. (**B**) Design of the SpG-BE4max sgRNA targeting the *LAMB2* p.E991K locus. The red nucleotide indicates the mutation site, and the green nucleotides indicate the PAM sequence. (**C**) Representative Sanger sequencing chromatograms of edited blastocysts. The highlighted position represents the edited nucleotide. (**D**) Representative F0 *LAMB2* p.E991K mutant rabbits and PCR genotyping results. (**E**) Sanger sequencing validation of the *LAMB2* mutation in newborn gene-edited rabbits. Red indicates the mutation site, green indicates the PAM sequence, and the underline indicates the sgRNA target region.

An evolutionary comparison of LAMB2 amino acid sequences among different mammalian species demonstrated full homology at the region surrounding the mutation site. The p.E987K missense mutation induces a polarity change by switching the highly retained glutamic acid (E) to lysine (K) (Figure 1A). To generate a rabbit model carrying the homologous *LAMB2* p.E991K mutation, a specific sgRNA targeting the corresponding locus was designed using the SpG-BE4max base-editing system (Figure 1B). Genotyping analysis performed at the blastocyst stage confirmed successful editing of the target site (Figure 1C). Following embryo microinjection and embryo transfer, founder (F0) gene-edited rabbits were successfully obtained (Figure 1D). Sanger sequencing further verified the presence of the intended nucleotide substitution at the target locus (Figure 1E), demonstrating the successful establishment of the *LAMB2* p.E991K mutant rabbit model.

### 3.2. High-Fat Diet Induction Caused Severe Dyslipidemia in LAMB2 p.E991K Rabbits

To investigate the effect of the *LAMB2* p.E991K mutation on lipid metabolism, 12-month-old mutant rabbits were subjected to a high-fat diet for four weeks. Four experimental groups were established, including WT-ND, WT-HFD, *LAMB2*^E991K^-ND, and *LAMB2*^E991K^-HFD. Blood samples were collected at weeks 2 and 4 for serum lipid analysis.

**Figure 2:**
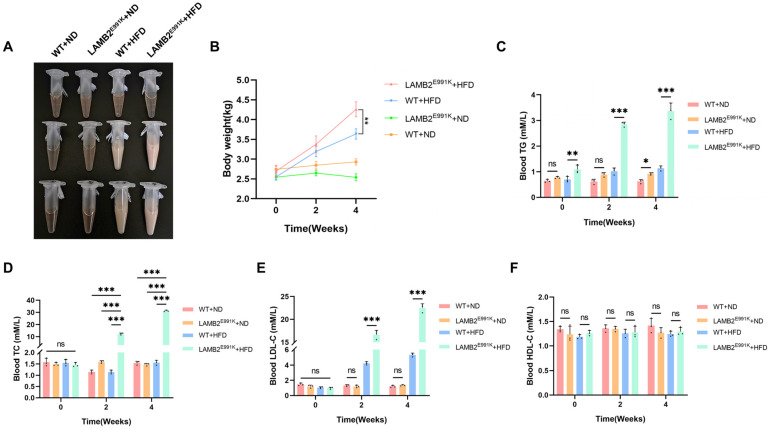
Changes in body weight and serum lipid profiles following high-fat diet induction. (**A**) Representative appearance of rabbit blood samples collected at weeks 0, 2, and 4. (**B**) Body weight changes in *LAMB2* p.E991K mutant rabbits and control groups following high-fat diet induction (*n* = 3). (**C**–**F**) Comparison of serum TC, TG, LDL-C, and HDL-C levels among different groups at weeks 0, 2, and 4 (*n* = 3). ns: Not significant, * *p* < 0.05, ** *p* < 0.01, and *** *p* < 0.001.

Prolonged high-fat diet consumption led to progressive, discernible alterations in the gross appearance of the blood. Compared with control groups, rabbits in the *LAMB2*^E991K^-HFD group exhibited a more pronounced milky appearance of the blood, which became progressively evident over time (Figure 2A), suggesting a substantial elevation in circulating lipid levels.

Further analysis revealed that rabbits in the *LAMB2*^E991K^-HFD group exhibited significantly increased body weight compared with the control groups (Figure 2B). Biochemical analysis revealed significant elevations in circulating TC, TG, and LDL-C concentrations in *LAMB2* mutant rabbits relative to WT animals, whereas HDL-C levels exhibited a non-significant upward trend. (Figure 2C–F). These findings indicate that the *LAMB2* p.E991K mutation markedly exacerbates lipid metabolic disorders under high-fat dietary conditions, suggesting an important role for LAMB2 in the maintenance of lipid homeostasis.

### 3.3. LAMB2 Mutation Accelerates Atherosclerotic Plaque Formation and Progression

**Figure 3:**
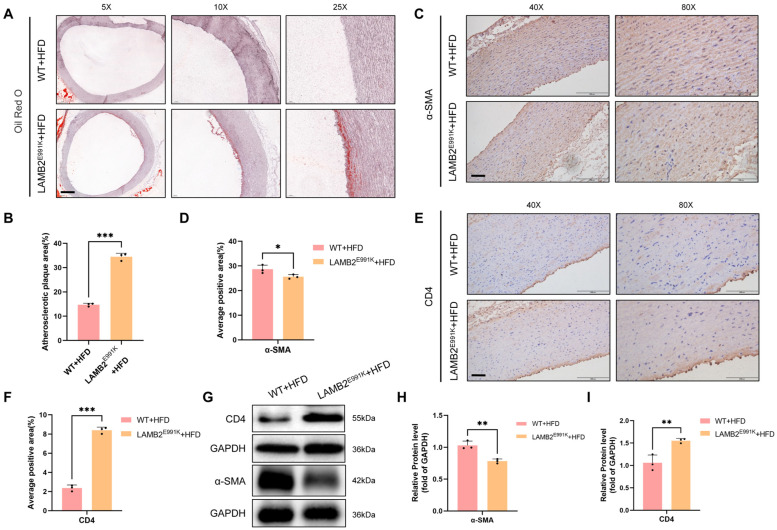
*LAMB2* mutations drive the development and progression of atherosclerotic plaques. (**A**) Oil Red O staining of aortic arch cross-sections. Scale bars: 500μm. (**B**) Quantification of plaque area in the aortic arch. (**C**) Immunohistochemical staining of α-SMA in the aortic arch. Scale bars: 200μm. (**D**) Quantitative analysis of α-SMA immunostaining. (**E**) Immunohistochemical staining of CD4-positive T cells in the aortic arch. Scale bars: 200μm. (**F**) Quantitative analysis of CD4 immunostaining. (**G**) Protein expression of α-SMA and CD4. (**H**) Quantification of α-SMA protein levels. (**I**) Quantification of CD4 protein levels. * *p* < 0.05, ** *p* < 0.01, and *** *p* < 0.001.

To determine whether *LAMB2* mutation promotes atherosclerotic plaque formation, aortic arch tissues from rabbits fed a high-fat diet were examined by Oil Red O staining. Plaque burden was quantified using ImageJ-based image analysis. Oil Red O staining revealed significantly increased lipid accumulation and plaque area in the *LAMB2* mutant group compared with the WT group (Figure 3A,B), indicating that the *LAMB2* mutation promotes lipid deposition and exacerbates atherosclerotic lesion formation.

To further assess plaque progression and stability, immunohistochemistry (IHC) and Western blot analyses were performed. Compared with WT rabbits, *LAMB2* mutant animals exhibited significantly lower α-SMA expression, as determined by IHC and Western blotting, indicating reduced vascular smooth muscle cell content within the vessel wall. (Figure 3C,D,G,H). Reduced α-SMA expression suggests a loss of smooth muscle cell content and impaired plaque stability. In contrast, CD4 expression was markedly increased in mutant rabbits (Figure 3E–G,I), indicating T-cell subset imbalance and enhanced inflammatory activity within the plaque microenvironment.

### 3.4. LAMB2 Mutation Disrupts Basement Membrane Integrity and Enhances Inflammatory Responses

**Figure 4:**
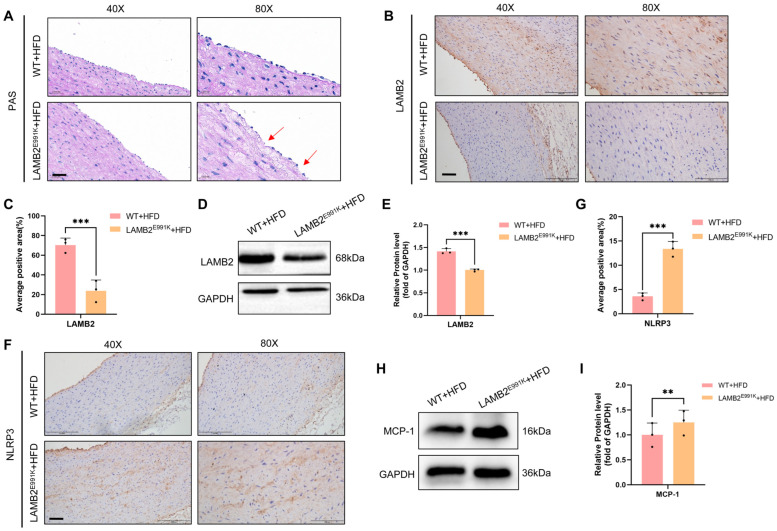
Basement membrane abnormalities and enhanced inflammation in the *LAMB2*^E991K^-HFD group. (**A**) PAS staining of the aortic arch. Arrows indicate regions with weakened basement membrane staining. Scale bars: 50μm. (**B**) Immunohistochemical staining of LAMB2 in the aortic arch. Scale bars: 200μm. (**C**) Quantitative analysis of LAMB2 immunostaining. (**D**) Protein expression of LAMB2. (**E**) Quantification of LAMB2 protein levels. (**F**) Immunohistochemical staining of NLRP3 in the aortic arch. Scale bars: 200μm. (**G**) Quantitative analysis of NLRP3 immunostaining. (**H**) Protein expression of MCP-1. (**I**) Quantification of MCP-1 protein levels. ** *p* < 0.01, and *** *p* < 0.001.

The vascular endothelial basement membrane serves as a critical structural barrier for maintaining vascular integrity and permeability. To evaluate basement membrane integrity, PAS staining was performed on aortic tissues. Compared with the WT group, the *LAMB2*^E991K^-HFD group exhibited weakened and discontinuous linear PAS-positive signals, indicating disruption of basement membrane structure (Figure 4A).

Consistent with these findings, both immunohistochemical staining and Western blot analysis demonstrated significantly reduced LAMB2 protein expression in the aortic tissues of mutant rabbits (Figure 4B–E). These pathological changes closely resemble the basement membrane abnormalities observed in Pierson syndrome caused by LAMB2 deficiency, suggesting that impaired LAMB2 expression similarly compromises endothelial function in the vascular system.

Because inflammation plays a central role in atherosclerosis progression, inflammatory markers were further evaluated. Via immunohistochemical mapping, we noted a robust upregulation of NLRP3 in the *LAMB2* mutant cohort (Figure 4F,G), a feature directly reflecting the stimulation of this specific inflammasome pathway. This observation aligns with historical data identifying the NLRP3 inflammasome cascade as a prime catalyst for rapid plaque advancement [20].

MCP-1 is a critical chemokine involved in inflammatory cell recruitment during atherosclerosis [21]. Western blot analysis showed increased MCP-1 expression in the *LAMB2* mutant group (Figure 4H,I), indicating enhanced inflammatory activation in mutant rabbits. Taken together, these findings demonstrate that the *LAMB2* p.E991K mutation exacerbates atherosclerotic progression through disruption of basement membrane integrity and amplification of inflammatory responses.

## 4. Discussion

As a major contributor to cardiovascular morbidity and mortality, atherosclerosis is a multifaceted disease involving complex interactions among lipid dysregulation, chronic vascular inflammation, and arterial remodeling. Despite the identification of numerous atherosclerosis-associated loci through GWAS, the biological relevance and mechanistic contributions of many candidate genes remain incompletely defined [22]. A large-scale GWAS conducted by Aragam et al. first identified the *LAMB2* variant rs34759087 as being significantly associated with the risk of coronary atherosclerotic disease; however, the underlying mechanisms remained unclear [12]. In the present study, we successfully generated a homologous *LAMB2* p.E991K mutant rabbit model using the SpG-BE4max base-editing system and systematically evaluated its atherosclerotic phenotype under high-fat diet conditions. To our knowledge, this study provides the first in vivo evidence demonstrating that LAMB2 dysfunction promotes the progression of atherosclerosis, thereby offering experimental support for previous GWAS findings and offering a stable large-animal model for translational medicine and drug screening.

Animal models are indispensable tools for investigating the mechanisms underlying human diseases [23]. Rabbits are widely used in cardiovascular research because their lipid metabolism shares important similarities with that of humans and they exhibit pronounced hyperlipidemic responses to high-fat diets [24,25]. In the present study, *LAMB2* p.E991K mutant rabbits developed significantly more severe dyslipidemia than wild-type controls following high-fat diet feeding, as evidenced by markedly elevated serum TC, TG, and LDL-C levels. This resembles the mixed dyslipidemia commonly observed in patients at high risk for atherosclerotic cardiovascular disease [26]. Nevertheless, further clinical and mechanistic studies are required to determine whether *LAMB2* variants are specifically associated with a distinct subtype of human dyslipidemia. Interestingly, *LAMB2* p.E991K mutant rabbits also exhibited increased body weight following high-fat diet feeding. However, the absence of a significant weight phenotype under normal dietary conditions suggests that *LAMB2* mutation alone is insufficient to induce weight gain. Instead, *LAMB2* dysfunction may act as a genetic modifier that enhances susceptibility to metabolic stress caused by excessive dietary lipid exposure. The increased body weight observed in mutant rabbits may therefore reflect aggravated diet-induced metabolic imbalance rather than a direct effect of *LAMB2* mutation on body weight regulation.

Previous studies have reported that patients with Pierson syndrome frequently exhibit lipid metabolic abnormalities and hyperlipidemia, and LAMB2 deficiency is recognized as the primary genetic cause of this syndrome [15,27]. These observations suggest that LAMB2 may participate not only in basement membrane maintenance but also in the regulation of lipid homeostasis. We speculate that LAMB2 dysfunction may impair lipoprotein transport, endothelial barrier function, or lipid clearance, thereby facilitating the accumulation of LDL particles within the vascular wall and accelerating atherosclerotic lesion development.

In addition to dyslipidemia, inflammation is a central driver of atherosclerosis progression [28,29,30]. In the present study, we observed significantly increased expression of MCP-1 and NLRP3, accompanied by enhanced CD4-positive cell infiltration in the aortas of *LAMB2* mutant rabbits, indicating activation of the vascular inflammatory microenvironment. MCP-1 is a key chemokine involved in monocyte recruitment and inflammatory cell infiltration during atherogenesis, and its upregulation may facilitate the accumulation of inflammatory cells within vascular lesions [21]. The NLRP3 inflammasome is widely recognized as a critical molecular link between cholesterol crystal deposition and inflammatory responses during atherogenesis. Upon activation, the NLRP3 inflammasome promotes the maturation and release of pro-inflammatory cytokines, particularly IL-1β and IL-18, thereby amplifying local inflammatory responses and accelerating plaque progression [31,32]. Previous studies have demonstrated that NLRP3 deficiency attenuates atherosclerotic lesion development [20]. Therefore, the elevated levels of MCP-1 and NLRP3 observed in the present study suggests that *LAMB2* mutation may promote atherosclerotic progression by enhancing inflammatory cell recruitment and inflammasome-mediated inflammatory activation. Furthermore, increased CD4 expression reflects activation of adaptive immune responses mediated by T lymphocytes, indicating that LAMB2 deficiency may disrupt local immune homeostasis and establish a persistent pro-inflammatory environment that favors atherosclerotic progression [33].

Another notable finding of this study was the significant reduction in α-SMA expression within the aortic lesions of *LAMB2* mutant rabbits. α-SMA is a well-established marker of vascular smooth muscle cells (VSMCs), which play essential roles in maintaining fibrous cap integrity and plaque stability [34]. Decreased α-SMA expression generally indicates either VSMC loss or phenotypic switching, both of which contribute to plaque destabilization [35]. Combined with the increased plaque area observed by Oil Red O staining, our findings suggest that *LAMB2* mutation not only promotes plaque formation but may also accelerate the transition toward a more vulnerable plaque phenotype, thereby increasing the risk of future plaque rupture and thrombotic events.

Beyond serving as a structural marker of VSMCs, α-SMA reduction may also reflect profound alterations in VSMC phenotype and function [35]. Increasing evidence suggests that extracellular matrix integrity and basement membrane composition play essential roles in maintaining the contractile phenotype of VSMCs [26]. Because LAMB2 is a key component of the vascular basement membrane, reduced LAMB2 expression may disrupt cell–matrix interactions and alter integrin-mediated signaling, thereby promoting VSMC phenotypic modulation from a contractile to a synthetic state. Synthetic VSMCs exhibit reduced α-SMA expression, impaired contractile capacity, and increased secretion of inflammatory mediators and extracellular matrix-remodeling enzymes, all of which contribute to plaque progression and instability. Although these mechanisms were not directly investigated in the present study, our findings suggest that LAMB2 dysfunction may represent an upstream regulator of VSMC homeostasis and provide a novel link between basement membrane abnormalities and vascular remodeling during atherosclerosis.

As a key component of the laminin family, the most fundamental biological function of LAMB2 is the maintenance of basement membrane integrity [36]. Therefore, we further investigated the effects of *LAMB2* mutation on vascular basement membrane structure. PAS staining revealed marked disruption of basement membrane continuity in the aortas of mutant rabbits, while immunohistochemistry and Western blot analyses confirmed a significant reduction in LAMB2 protein expression. These findings are highly consistent with the pathological characteristics observed in the glomerular basement membrane defects of Pierson syndrome, suggesting that LAMB2 deficiency exerts similar detrimental effects on basement membranes in different tissues. The vascular basement membrane is essential for preserving endothelial barrier function, and its disruption increases vascular permeability, facilitating the infiltration of circulating lipoproteins and inflammatory cells into the vessel wall [37,38]. Therefore, we propose that basement membrane injury may represent a critical initiating event in *LAMB2*-mediated atherogenesis. Reduced basement membrane integrity resulting from *LAMB2* mutation may subsequently trigger lipid accumulation, inflammatory cell recruitment, endothelial dysfunction, and ultimately the development of atherosclerotic lesions.

A plausible mechanism emerging from the present study is that the *LAMB2* p.E991K mutation decreases LAMB2 abundance, resulting in basement membrane dysfunction and impaired vascular barrier integrity. Increased endothelial permeability may facilitate immune cell entry into the vessel wall and potentiate inflammatory responses, promoting NLRP3 inflammasome activation and arterial lipoprotein accumulation. Simultaneously, disruption of vascular smooth muscle cell homeostasis may weaken plaque stability. Through the combined effects of vascular barrier dysfunction, inflammation, lipid retention, and smooth muscle cell loss, *LAMB2* mutation may accelerate atherosclerotic lesion development.

Despite the strengths of the present study, several limitations should be considered. The relatively limited sample size may have reduced the robustness of certain statistical analyses. In addition, our work primarily characterized pathological alterations and protein expression changes, whereas the intracellular signaling networks associated with LAMB2 dysfunction remain insufficiently defined. Moreover, although the high-fat diet-fed rabbit model reproduces key features of atherosclerosis, it does not entirely reflect the prolonged and multifactorial progression of human disease. Future investigations combining transcriptomic profiling, single-cell sequencing, and endothelial cell-based functional studies are needed to further clarify the molecular pathways through which LAMB2 influences vascular integrity and inflammatory regulation. Elucidating these mechanisms may also provide insights into the feasibility of LAMB2-targeted therapeutic strategies for atherosclerotic cardiovascular disease.

## 5. Conclusions

In summary, our study provides the first in vivo evidence that the *LAMB2* p.E991K mutation increases susceptibility to high-fat diet-induced atherosclerosis. The mutation promotes atherosclerotic progression through lipid metabolic disturbances, basement membrane dysfunction, and inflammatory activation. These findings highlight the critical role of LAMB2 in vascular homeostasis and reveal how genetic predisposition interacts with environmental metabolic stress to influence atherosclerosis susceptibility. From a practical perspective, *LAMB2* genetic variation may serve as a potential marker for identifying individuals with increased susceptibility to atherosclerotic cardiovascular disease, while targeting LAMB2-associated vascular dysfunction may represent a potential therapeutic strategy that warrants further investigation.

## Data Availability

The original contributions presented in this study are included in the article material. Further inquiries can be directed to the corresponding author.

## Abbreviations

The following abbreviations are used in this manuscript:

GWAS	genome-wide association study
LAMB2	Laminin β2
WT-ND	wild-type normal diet
*LAMB2*^E991K^-ND	*LAMB2* normal diet
WT-HFD	wild-type high-fat diet
*LAMB2*^E991K^-HFD	*LAMB2* high-fat diet
TC	total cholesterol
TG	triglycerides
HDL-C	high-density lipoprotein cholesterol
LDL-C	low-density lipoprotein cholesterol
IHC	Immunohistochemical
